# Glucose starvation induces NADPH collapse and disulfide stress in SLC7A11^high^ cancer cells

**DOI:** 10.18632/oncotarget.27993

**Published:** 2021-08-03

**Authors:** Xiaoguang Liu, Boyi Gan

**Keywords:** SLC7A11, cystine, cysteine, NADPH, glucose starvation

Malignant cells are known to exhibit increased glucose uptake compared to normal cells. Besides providing energy “currency” ATP, glucose also contributes to intracellular redox maintenance through generating the universal cellular reduction “currency” reduced nicotinamide adenine dinucleotide phosphate (NADPH) primarily via the pentose phosphate pathway (PPP), as well as donates carbon intermediates for the biosynthesis of diverse macromolecules. Glucose starvation induces rapid cell death in some cancer cell lines whereas other cancer cell lines are resistant to glucose deprivation. However, the genetic determinants underlying differential sensitivities to glucose starvation–induced cell death in cancer cells remain incompletely understood.

In 2017, several groups independently identified solute carrier family 7 member 11 (SLC7A11; also known as xCT) as a key determinant of cell death under glucose deprivation in a wide variety of cancer cell lines, revealing that cancer cells with high expression of SLC7A11 (SLC7A11^high^) are much more sensitive to glucose deprivation–induced cell death than SLC7A11^low^ cancer cells [1–3]. SLC7A11 and solute carrier family 3 member 2 (SLC3A2) form the system x_c_^−^, a sodium-independent antiporter that imports extracellular cystine and exports intracellular glutamate. In this transporter complex, SLC7A11 is responsible for the primary cystine transporting activity and substrate specificity, whereas SLC3A2 mainly serves as a chaperone protein for SLC7A11 [4–6]. Extracellular cystine is transported into cells primarily through SLC7A11 and then is quickly reduced to cysteine, which serves as the limiting precursor for the synthesis of reduced glutathione (GSH), a major intracellular antioxidant [4]. SLC7A11-mediated cystine uptake and GSH biosynthesis has a well-known role in suppressing oxidative stress–induced cell death such as ferroptotic cell death [6, 7]; therefore, the pro-cell death role of SLC7A11 under glucose starvation was rather surprising.

Recent studies provide novel insights into these seemly counterintuitive observations. Specifically, cystine is one of the least soluble amino acids, and cystine accumulation in cytosol upon its uptake by SLC7A11 can be highly toxic to SLC7A11^high^ cancer cells, forcing such cells to quickly reduce cystine to much more soluble cysteine. This high rate of cystine reduction to cysteine requires a large amount of NADPH, which is mainly supplied from glucose through the PPP, thereby inducing glucose- and PPP-dependency in SLC7A11^high^ cancer cells [8, 9]. Consequently, glucose starvation, by limiting NADPH production, leads to a marked accumulation of intracellular cystine and other disulfide molecules such as glutathione disulfide (GSSG), which is accompanied with reactive oxygen species (ROS) accumulation, GSH and NADPH depletion, and rapid cell death in SLC7A11^high^ cancer cells [8]. It’s well known that aberrant ROS accumulation is toxic to cell and can induce cell death. Unexpectedly, ROS scavengers Trolox or Tempol, despite effectively quenching ROS, exerted little rescuing effect on cystine accumulation, NADPH depletion, or cell death in SLC7A11^high^ cancer cells under glucose starvation; in contrast, these redox defects and cell death can be readily rescued by treatments that prevent disulfide accumulation, such as penicillamine and TCEP [8]. These data suggest that the cell death in SLC7A11^high^ cancer cells under glucose starvation is likely caused by intracellular disulfide accumulation and NADPH depletion, but not by ROS *per se*. Therefore, while SLC7A11^high^ cancer cells have stronger antioxidant capabilities and can survive and grow better under oxidative stress conditions, high cystine uptake in SLC7A11^high^ cancer cells also exposes a metabolic liability and renders these cells to be exquisitely vulnerable under glucose limiting conditions. Our very recent study further showed that *KEAP1*-mutant lung cancer cells or tumors (which exhibit aberrant SLC7A11 expression due to constitutive activation of NRF2 transcription factor) are also more dependent on glucose for survival and more sensitive to glucose transporter (GLUT) inhibition than their wild-type counterparts, suggesting a therapeutic strategy to target this largely incurable cancer subtype [10].

These studies also open up several questions for future investigations. Exactly how disulfide stress (cellular stress that is induced by aberrantly accumulation of intracellular cystine and other disulfide molecules) and NADPH depletion induce cell death remains elusive. Crystallization of insoluble cystine either in the bladder (cystinuria) or intracellular lysosomes (cystinosis) is known to be toxic to cells or organs [11, 12]. Further studies are required to determine whether cystine crystals could be detected in SLC7A11^high^ cancer cells under glucose deprivation. In addition, recently we found that glucose starvation in SLC7A11^high^ cancer cells suppressed histone 2A ubiquitination (H2Aub) likely through NADPH depletion and subsequent phosphorylation and inhibition of BMI1, a critical subunit of polycomb-repressive complex 1 (PRC1) that mediates H2Aub in nucleus, leading to transcription upregulation of genes involved in endoplasmic reticulum (ER) stress response and subsequent cell death [13]. Importantly, treatment with 2-deoxy-D-glucose (2DG, which can be shunted into the PPP to generate NADPH) largely rescued the effects of glucose starvation on BMI1 phosphorylation, H2Aub levels, and ER stress under glucose starvation [13]. Therefore, this study supports a critical role of NADPH depletion (and possibly disulfide stress) in mediating glucose starvation–induced BMI1 phosphroylation and H2Aub reduction, suggesting that disulfide stress can modulate downstream cellular signaling in SLC7A11^high^ cancer cells. It is conceivable that the activities of upstream kinases or phosphatases of BMI1 may be regulated by disulfide stress (through posttranslational modifications) or NADPH (which might act as a cofactor for these enzymes). Future studies will be directed to test this intriguing hypothesis. Finally, the reductase(s) that mediates the reduction of cystine to cysteine remains elusive. Glutathione reductase (GR) and cytosolic thioredoxin reductase 1 (TR1) are the presumed cystine reductases. Thioredoxin-related protein of 14 kDa (TRP14) has also been suggested as a cystine reductase based its ability to catalyze cystine reduction *in vitro* [14]. However, the definitive biological evidence that genetic ablation of any of these putative cystine reductases would result in aberrant accumulation of intracellular cystine is still lacking. Identification and characterization of cystine reductase(s) will provide additional mechanistic insights on redox maintenance in SLC7A11^high^ cancer cells.
